# Missing continuous outcomes under covariate dependent missingness in cluster randomised trials

**DOI:** 10.1177/0962280216648357

**Published:** 2016-05-13

**Authors:** Anower Hossain, Karla Diaz-Ordaz, Jonathan W Bartlett

**Affiliations:** 1 Department of Medical Statistics, London School of Hygiene & Tropical Medicine (LSHTM), London, UK; 2 Statistical Innovation Group, AstraZeneca, Cambridge, UK

**Keywords:** Cluster randomised trials, missing outcome data, covariate dependent missingness, multiple imputation, complete records analysis

## Abstract

Attrition is a common occurrence in cluster randomised trials which leads to missing outcome data. Two approaches for analysing such trials are cluster-level analysis and individual-level analysis. This paper compares the performance of unadjusted cluster-level analysis, baseline covariate adjusted cluster-level analysis and linear mixed model analysis, under baseline covariate dependent missingness in continuous outcomes, in terms of bias, average estimated standard error and coverage probability. The methods of complete records analysis and multiple imputation are used to handle the missing outcome data. We considered four scenarios, with the missingness mechanism and baseline covariate effect on outcome either the same or different between intervention groups. We show that both unadjusted cluster-level analysis and baseline covariate adjusted cluster-level analysis give unbiased estimates of the intervention effect only if both intervention groups have the same missingness mechanisms and there is no interaction between baseline covariate and intervention group. Linear mixed model and multiple imputation give unbiased estimates under all four considered scenarios, provided that an interaction of intervention and baseline covariate is included in the model when appropriate. Cluster mean imputation has been proposed as a valid approach for handling missing outcomes in cluster randomised trials. We show that cluster mean imputation only gives unbiased estimates when missingness mechanism is the same between the intervention groups and there is no interaction between baseline covariate and intervention group. Multiple imputation shows overcoverage for small number of clusters in each intervention group.

## 1 Introduction

In cluster randomised trials (CRTs), identifiable clusters of individuals such as villages, schools, medical practices – rather than individuals – are randomly allocated to each of intervention and control groups, while individual-level outcomes of interest are observed within each cluster. The number of clusters and/or the cluster sizes in each intervention group might be different. CRTs with equal number of clusters in each intervention group with constant cluster size are known as balanced CRTs. One important characteristic of CRTs is that the outcomes of individuals within the same cluster may exhibit more similarity compared to the outcomes of individuals in the other clusters, which is quantified by the intraclass correlation coefficient (ICC), denoted by *ρ*. In practice, the value of ICC typically ranges from 0.001 to 0.05 and it is rare for clinical outcomes to have ICC above 0.1.^1^ Small values of ICC can lead to substantial variance inflation factors and should not be ignored.^2,3^ CRTs are being increasingly used in the fields of health promotion and health service research. Reasons for such popularity include the nature of intervention that itself may dictate its application at the cluster level, less risk of intervention contamination and administrative convenience.^4^ It is well known that the power and precision of CRTs are lower relative to trials that individually randomise the same number of individuals.^2^ In spite of this, the advantages associated with CRTs are perceived by researchers to outweigh the potential loss of statistical power and precision in some situations.

Attrition is a common problem for CRTs, leading to missing outcome data. This not only reduces the statistical power of the study but may result in biased intervention effect estimates.^5^ Handling missing data in CRTs is complicated by the fact that data are clustered. Inadequate handling of the missing data may result in misleading inferences.^6^ A systematic review^7^ revealed that, among all CRTs published in English in 2011, 72% of trials had missing values either in outcomes or in covariates or in both. Among them only 34% of CRTs reported how they handled missing data. One of the reasons may be that the methodological development for dealing with missing data in CRTs has been relatively slow in spite of the increasing popularity of CRTs. Cluster mean imputation has been suggested as a valid approach for handling missing outcome data in CRTs.^8^

The impact of missing data on estimation and inference of a parameter of interest depends on the missing data mechanism, the method used to handle the missing data, and the choice of statistical methods used for data analysis. In this paper, we study the validity of three analysis methods – unadjusted cluster-level analysis, adjusted cluster-level analysis and linear mixed model (LMM) – when there is missingness in the continuous outcome, and this missingness depends on baseline covariates, and conditional on these baseline covariates, not on the outcomes itself. We compare the performance of these methods on complete records and multiply imputed datasets. In addition, we investigate the validity of cluster mean imputation, as proposed by Taljaard et al.,^8^ under the same missingness assumption.

This paper is organised as follows. Section 2 presents a brief review of the approaches to the analysis of CRTs with complete data. In Section 3, the assumed missingness mechanism for CRTs is described. Section 4 describes methods of handling missing data in CRTs. In Section 5, we investigate the validity of complete records analysis of CRTs. Section 6 describes a simulation study and presents the results. We conclude the study with some discussion in Section 7.

## 2 Analysis of CRTs with complete data

We begin by describing the two broad approaches to the analysis of CRTs in the absence of missing data. These are cluster-level analysis and individual-level analysis.

### 2.1 Cluster-level analysis

Cluster-level analysis can be done in two ways: unadjusted cluster-level analysis and baseline covariate adjusted cluster-level analysis. This approach can be explained as a two-stage process. In the first stage of unadjusted analysis, a relevant summary measure of outcomes is calculated for each cluster. Then, in the second stage, the cluster-specific summary measures of the control and intervention groups obtained in the first stage are compared using appropriate statistical methods. The most common one is the standard *t*-test for two independent samples (here referred to as cluster-level *t*-test) with degrees of freedom (DF) equal to the total number of clusters in the study minus two. The basis of using this test is that the resulting summary measures are statistically independent, which is a consequence of the clusters being independent of each other. In the case of baseline covariate adjusted analysis, an individual-level regression analysis is carried out at the first stage including all covariates as explanatory variables, except for the intervention indicator, and ignoring the clustering of the data.^4,9^ The individual level residuals from the first-stage model are then used to calculate the cluster-specific summary measures for the control group and the intervention group, which are then compared using cluster-level *t*-test in the second stage of analysis to evaluate the intervention effect adjusted for baseline covariates. The main purposes of adjusting for baseline covariates are to increase the credibility of the trial findings by demonstrating that any observed intervention effect is not attributed to the possible imbalance between the intervention groups in term of baseline covariates and to improve the statistical power.^10^

### 2.2 Individual-level analysis

In individual-level analysis, a regression model is fitted to the individual-level outcomes, allowing for the fact that observations within the same cluster are correlated. LMM is widely used as individual-level analysis for CRTs with continuous outcomes. The LMM takes into account between-cluster variability using cluster-level effects which are assumed to follow a specified probability distribution. The parameters of that distribution are estimated using maximum likelihood methods together with intervention effect and other covariates effects. Generalised estimating equations are an alternative approach, but for continuous outcomes and an exchangeable correlation matrix, estimates are identical to those from LMM with a random intercept.^11^

The adjusted *t*-test, proposed by Donner and Klar,^2^ is an alternative approach to test the intervention effect for quantitative outcomes, which involves calculating the mean of the individual outcome values in each intervention group. These means are then compared using a *t*-test in which the standard error (SE) is adjusted to account for the intracluster correlation. The adjusted *t*-test and the cluster-level *t*-test are identical for balanced CRTs.

## 3 Missingness mechanism assumptions for CRTs

In this paper, we will consider the common setting where the outcomes are continuous, and only outcomes are missing. In statistical analysis, if there are missing values, an assumption must be made about the missingness mechanism, which refers to the relationship between missingness and the underlying values of the variables in the data.^12^ According to Rubin’s framework,^13^ a missingness mechanism can be classified as (i) missing completely at random (MCAR), where the probability of a value being missing is independent of the observed and unobserved data; (ii) missing at random (MAR), where conditioning on the observed data, the probability of a value being missing is independent of the unobserved data; and (iii) missing not at random (MNAR), where the probability of value being missing depends on both observed and unobserved data.

In CRTs, an assumption that may sometimes be plausible is that missingness in outcomes depends on covariates measured at baseline and conditional on these baseline covariates, not on the outcome itself. We refer to this as covariate dependent missingness (CDM). For example, blood pressure outcome data could be CDM if missingness in blood pressure measurement depends on covariates (e.g. age, BMI or weight), but given these, not on the blood pressure measurement itself. CDM is an example of a MAR mechanism when covariates are fully observed.

Let *Y_ijl_* be a continuous outcome of interest for the *l*th $\left(l=1,2,\ldots ,m_{\mathrm{ij}}\right)$ individual in the *j*th $\left(j=1,2,\ldots ,k_{i}\right)$ cluster of the intervention group *i* (i=1,2), where *i* = 1 corresponds to control group and *i* = 2 corresponds to intervention group. We assume that the *Y_ijl_* follow a LMM given by

(1) $$Y_{\mathrm{ijl}}=\alpha_{i}+\beta_{i}X_{\mathrm{ijl}}+\delta_{\mathrm{ij}}+\varepsilon_{\mathrm{ijl}}$$

where *α_i_* is a constant for *i*th intervention group, *X_ijl_* is a baseline covariate value for (ijl)th individual, *β_i_* is the effect of baseline covariate *X* on *Y* in intervention group *i*, *δ_ij_* is the (ij)th cluster effect and *ε_ijl_* is the individual error term. We also assume that the cluster effect $\left(\delta_{\mathrm{ij}}\right)$ and the individual error $\left(\varepsilon_{\mathrm{ijl}}\right)$ are statistically independent, and $\text{E}(\delta_{\mathrm{ij}})=0,\text{Var}(\delta_{\mathrm{ij}})=\sigma_{b}^{2}$ and $\text{E}(\varepsilon_{\mathrm{ijl}})=0,\text{Var}(\varepsilon_{\mathrm{ijl}})=\sigma_{w}^{2}$, where $\sigma_{b}^{2}$ and $\sigma_{w}^{2}$ are the between-cluster variance and within-cluster variance, respectively. Later we will sometimes make normality assumptions on these random effects/random errors. Suppose the baseline covariate *X* has mean *μ_x_*. Then

$$\text{E}({\bar{Y}}_{i})=\alpha_{i}+\beta_{i}\mu_{x}=\mu_{i}$$

where ${\bar{Y}}_{i}=(1/k_{i})\sum_{j=1}^{k_{i}}(1/m_{\mathrm{ij}})\sum_{l=1}^{m_{\mathrm{ij}}}Y_{\mathrm{ijl}}=(1/k_{i})\sum_{j=1}^{k_{i}}{\bar{Y}}_{\mathrm{ij}}$. Here, ${\bar{Y}}_{i}$ and ${\bar{Y}}_{\mathrm{ij}}$ are the mean outcome of the *i*th intervention group and the (ij)th cluster, respectively. With complete data, the cluster-level analysis estimate of the intervention effect, say $\hat{\theta }$, is then calculated as

$$\hat{\theta }={\bar{Y}}_{1}-{\bar{Y}}_{2}$$

With complete data, this estimator is unbiased for the true intervention effect, that is

$$\text{E}(\hat{\theta })=\mu_{1}-\mu_{2}$$

Suppose there are some missing values for outcome *Y*. Define a missing data indicator *R_ijl_* such that

$$R_{ijl}=\{\begin{array}{c} 1,\;\text{if}\;Y_{ijl}\;\text{is observed}\;\\ 0,\;\text{if}\;Y_{ijl}\;\text{is missing}\; \end{array}$$

Then $\sum_{l=1}^{m_{\mathrm{ij}}}R_{\mathrm{ijl}}$ is the number of observed outcomes in the (ij)th cluster. The CDM assumption can then be expressed as

$$P(R_{\mathrm{ijl}}=0|Y_{\mathrm{ij}},X_{\mathrm{ij}})=P(R_{\mathrm{ijl}}=0|X_{\mathrm{ijl}})$$

where $Y_{\mathrm{ij}}=(Y_{\mathrm{ij}1},Y_{\mathrm{ij}2},\ldots ,Y_{{\mathrm{ijm}}_{\mathrm{ij}}})$ and $X_{\mathrm{ij}}=(X_{\mathrm{ij}1},X_{\mathrm{ij}2},\ldots ,X_{{\mathrm{ijm}}_{\mathrm{ij}}})$ are the vectors of the outcomes and the baseline covariate values, respectively, in the (ij)th cluster. In other words, the missingness of the (ijl)th individual’s outcome *Y_ijl_* depends only on that individual’s baseline covariate value *X_ijl_*.

## 4 Methods of handling missing data in CRTs

Common approaches for handling missing data in CRTs include complete records analysis (CRA), single imputation and multiple imputation (MI). This section describes these approaches. In this paper, we focused on CRA and MI since they are the most commonly used methods for handling missing data.

### 4.1 CRA

In CRA, often referred to as complete case analysis, only individuals with outcome observed are considered in the analysis, while individuals with missing outcome are excluded. It is widely used because of its simplicity and is usually the default method of most statistical packages. It is well known that CRA is valid if data are MCAR or if missingness is independent of the outcome, conditional on covariates.^12^ Likelihood-based CRA is valid under MAR, if missingness is only in the outcome and all predictors of missingness are conditioned on in the model.^12^ CRA is also valid under MNAR mechanisms where missingness in a covariate is dependent on the value of that covariate, but is conditionally independent of outcome.^14,15^

### 4.2 Single imputation

Single imputation imputes a single value for each missing outcome and creates a complete dataset. In general, single imputation is not recommended, since estimates of uncertainty are biased downwards, leading to anti-conservative inferences. However, for CRTs two choices for single imputation are group mean imputation and cluster mean imputation.^8^ In the first case, missing outcomes in each intervention group are replaced by the mean outcome calculated using complete records pooled across clusters of that group. This approach reduces the variability among the clusters means and, therefore, gives inflated Type I error.^8^ In cluster mean imputation, missing outcomes in each cluster are replaced by the mean outcome calculated using complete records of that cluster. This approach has been suggested as a good approach for handling missing outcomes by Taljaard et al.^8^ They showed that cluster mean imputation gives Type I error close to nominal level under MCAR, using adjusted *t*-test with balanced CRTs. However, under MAR or CDM, adjusted *t*-test with cluster mean imputation may not be valid. We note that, with balanced CRTs, the cluster-level *t*-test and the adjusted *t*-test are identical with cluster mean imputation since after imputation the cluster sizes become constant and the cluster means remain unchanged by the imputation. Consequently, our later results for the validity of cluster level t-test can also be applied to infer the validity of results after using cluster mean imputation. One additional problem with cluster mean imputation is that it distorts the estimates of between-cluster variability and within-cluster variability, which often are of interest.

### 4.3 MI

MI, first proposed by Rubin,^16^ is a method of filling in the missing outcomes multiple times by simulating from an appropriate model. The aim of imputing multiple times is to allow for the uncertainty about the missing outcomes due to the fact that the imputed values are sampled draws for the missing outcomes. A sequence of *Q* imputed datasets is obtained by replacing each missing outcome by a set of Q≥2 imputed values that are simulated from an appropriate distribution or model. Each of the *Q* datasets is then analysed as a completed dataset using a standard method. The results from the *Q* imputed datasets are then combined using Rubin’s rules.^16^ The combined inference is based on a *t*-distribution with DF given by

(2) $$\nu =(Q-1)(1+\frac{Q}{Q+1}\frac{W_{\text{MI}}}{B_{\text{MI}}}{)}^{2}$$

where $B_{\text{MI}}$ is the between-imputation variance and $W_{\text{MI}}$ is the average within-imputation variance. This formula for DF is derived under the assumption that the complete data DF, $\nu_{\text{com}}$, is infinite.^17^

In CRTs, $\nu_{\text{com}}$ is usually small as it is based on the number of clusters in each intervention group rather than the number of individuals. For unadjusted cluster-level analysis and individual-level baseline covariate adjusted cluster-level analysis, $\nu_{\text{com}}$ is calculated as $k_{1}+k_{2}-2$ for statistical inference using cluster-level *t*-test^4^ and adjusted *t*-test.^8^ An adjustment is made to the $\nu_{\text{com}}$ to adjust for cluster-level baseline covariates using cluster-level analysis. In this case, we reduce the complete data DF from $\nu_{\text{com}}=k_{1}+k_{2}-2$ to $\nu_{\text{com}}=k_{1}+k_{2}-2-p$, where *p* is the number of parameters corresponding to the cluster-level baseline covariates in the first-stage regression model.^4^

When $\nu_{\text{com}}$ is small and there is a modest proportion of missing data, the repeated-imputation DF, *ν* (given in equation (2)), for reference *t*-distribution can be much higher than $\nu_{\text{com}}$, which is not appropriate.^17^ In such a situation, a more appropriate DF, $\nu_{\text{adj}}$, proposed by Barnard and Rubin,^17^ is calculated as

(3) $$\nu_{\text{adj}}={\left(\frac{1}{\nu }+\frac{1}{{\hat{\nu }}_{\text{obs}}}\right)}^{-1}\leq \nu_{\text{com}}$$

where

(4) $${\hat{\nu }}_{\text{obs}}={\left(1+\frac{Q+1}{Q}\frac{B_{\text{MI}}}{W_{\text{MI}}}\right)}^{-1}\left(\frac{\nu_{\text{com}}+1}{\nu_{\text{com}}+3}\right)\nu_{\text{com}}$$

At least four different types of MI have been used in CRTs.^7^ These are *standard* MI which ignores clustering, *fixed effects* MI which includes a fixed effect for each cluster in the imputation model, *random effects* MI where clustering is taken into account through random effects in the imputation model and *within-cluster* MI where standard MI is applied within each cluster. Andridge^18^ showed, with balanced CRTs under MCAR and MAR missingness in a continuous outcome with a single covariate in addition to intervention indicator, that MI models that incorporate clustering using fixed effects for cluster can result in a serious overestimation of variance of group means and this overestimation is more serious for small cluster sizes and small ICCs. This overestimation of variance results in a decrease in power, which is particularly dangerous for CRTs which are often underpowered.^18^ MI using random effects for cluster gave slight overestimation of variance of group means for very small values of *ρ*. Andridge also showed that using an MI model that ignores clustering can lead to severe underestimation of the MI variance for large values of ρ (>0.005). This underestimation of variance leads to inflated Type I error.

Taljaard et al.^8^ examined the performance of MI in a simple setup considering balanced CRTs where there are no covariates except intervention indicator using standard regression imputation, which ignores clustering, and random effects MI which does account for intraclass correlation. They also considered the Approximate Bayesian Bootstrap (ABB) procedure, proposed by Rubin and Schenker,^19^ as a non-parametric MI. In ABB, sampling from the posterior predictive distribution of missing data is approximated by first generating a set of plausible contributors drawn with replacement from the observed data, and then imputed values are drawn with replacement from the possible contributors. Two possible uses of ABB in CRTs are pooled ABB and within-cluster ABB, where the set of possible contributors are sampled from all observed values across the clusters in each group or from observed values in the same cluster, respectively. They showed that none of these four MI procedures tend to yield better power compared to the power of adjusted *t*-test using no imputation and cluster mean imputation under MCAR.

We note that in the case of missing outcome under MAR for individually randomised trials, Groenwold et al.^20^ showed that CRA with covariate adjustment and MI give similar estimates so long as the same set of predictors of missingness is used. It can be anticipated that similar result holds for CRTs. An obvious advantage of CRA over MI is that it is much easier to apply, and therefore in situations where they are equivalent, CRA is clearly preferable.

## 5 Validity of CRA of CRTs

In this section, we describe the unadjusted cluster-level analysis, baseline covariate adjusted cluster-level analysis and LMM analysis methods using complete records, and derive conditions under which they give valid inferences under the CDM assumption.

### 5.1 Unadjusted cluster-level analysis using complete records

The mean of the observed outcomes in the *i*th intervention group can be calculated as

$${\bar{Y}}_{i}^{\text{obs}}=\frac{1}{k_{i}}\sum_{j=1}^{k_{i}}{\bar{Y}}_{ij}^{\text{obs}}$$

where ${\bar{Y}}_{ij}^{\text{obs}}=\left(1/\sum_{l=1}^{m_{ij}}R_{ijl}\right)\sum_{l=1}^{m_{ij}}R_{ijl}Y_{ijl}$ is the observed mean of (ij) th cluster. The estimate of intervention effect is given by

(5) $${\hat{\theta }}^{\text{obs}}={\bar{Y}}_{1}^{\text{obs}}-{\bar{Y}}_{2}^{\text{obs}}$$

In Appendix 1, we show that

(6) $$\text{E}\left({\hat{\theta }}^{\text{obs}}\right)=\mu_{1}-\mu_{2}+\beta_{1}\left(\mu_{x11}-\mu_{x}\right)-\beta_{2}\left(\mu_{x21}-\mu_{x}\right)$$

and

(7) $$\text{Var}\left({\hat{\theta }}^{\text{obs}}\right)=\sum_{i=1}^{2}\frac{1}{k_{i}}\left(\beta_{i}^{2}\sigma_{{\bar{x}}_{i1}}^{2}+\sigma_{b}^{2}+\frac{\sigma_{w}^{2}}{\eta_{i}}\right)$$

where $\mu_{\mathrm{xi}1}$ is true mean of the baseline covariate *X* in the *i*th intervention group among those individuals with observed outcomes, $\sigma_{{x¯}_{i1}}^{2}$ is the variance of the cluster-specific means of *X* among those with observed outcomes and $1/\eta_{i}=\text{E}(1/\sum_{l}R_{\mathrm{ijl}})$. From equation (6), it follows that the unadjusted cluster-level analysis using CRA will be unbiased if

(8) $$\beta_{1}(\mu_{x11}-\mu_{x})=\beta_{2}(\mu_{x21}-\mu_{x}),\text{or equivalently},\frac{\beta_{1}}{\beta_{2}}=\frac{\mu_{x21}-\mu_{x}}{\mu_{x11}-\mu_{x}}$$

A sufficient condition for equation (8) to hold is that $\beta_{1}=\beta_{2}$ (i.e. there is no interaction between baseline covariate and intervention group in the outcome model) and that the missingness mechanisms are the same in the two intervention groups, so that $\mu_{x11}=\mu_{x21}$. It can also be seen from equation (6) that, when there is no missing data, $\mu_{x11}=\mu_{x21}=\mu_{x}$, and hence the unadjusted cluster-level analysis results in unbiased estimates of intervention effects even when $\beta_{1}\neq \beta_{2}$.

### 5.2 Adjusted cluster-level analysis using complete records

Recall that the first step of the adjusted cluster-level analysis involves fitting a regression model for Y with X as covariate, but ignoring the intervention indicator and clustering of the data. The residual ${\hat{\epsilon }}_{ijl}$ is then given by

$${\hat{\epsilon }}_{ijl}=Y_{ijl}-{\hat{Y}}_{ijl}$$

where ${\hat{Y}}_{ijl}=\gamma +\lambda X_{ijl}$ is the predicted outcome for the (ijl)th individual based on the first-stage model fit. The mean of the observed residuals of the *i*th group is given by

$${\bar{\hat{\epsilon }}}_{i}^{\text{obs}}=\frac{1}{k_{i}}\sum_{j=1}^{k_{i}}{\bar{\hat{\epsilon }}}_{\mathrm{ij}}^{\text{obs}}$$

where ${\bar{\hat{\epsilon }}}_{\mathrm{ij}}^{\text{obs}}=1/\left(\sum_{l=1}^{m_{ij}}R_{ijl}\right)\sum_{l=1}^{m_{ij}}R_{ijl}{\hat{\epsilon }}_{ijl}$ is the mean of observed residuals of the (ij)th cluster. The baseline covariate adjusted estimator of intervention effect is given by

(9) $${\hat{\theta }}_{\text{adj}}^{\text{obs}}={\bar{\hat{\epsilon }}}_{1}^{\text{obs}}-{\bar{\hat{\epsilon }}}_{2}^{\text{obs}}$$

We show in Appendix 2 that

(10) $$\text{E}\left({\hat{\theta }}_{\text{adj}}^{\text{obs}}\right)=\mu_{1}-\mu_{2}+\beta_{1}\left(\mu_{x11}-\mu_{x}\right)-\beta_{2}\left(\mu_{x21}-\mu_{x}\right)+\lambda \left(\mu_{x21}-\mu_{x11}\right)$$

Hence, the estimator (9) will be unbiased if (i) $\beta_{1}=\beta_{2}$ and $\mu_{x11}=\mu_{x21}$, or if (ii) $\lambda =\beta_{1}=\beta_{2}$. Equation (10) is derived (see Appendix 2) assuming fixed values of γ and λ instead of their estimates. In practice, *γ* and *λ* are unknown and must be estimated by fitting the first-stage regression model for the observed outcomes. We are not worried about the estimate of the intercept parameter *γ* since the expression (10) is independent of *γ*. If *λ* is estimated consistently, then ${\hat{\theta }}_{\text{adj}}^{\text{obs}}$ will be a consistent estimator of intervention effect when in truth $\lambda =\beta_{1}=\beta_{2}$. The estimator of *λ*, say $\hat{\lambda }$, is calculated using complete records and will be unbiased (and therefore consistent) if $R_{\mathrm{ijl}}⊥⊥Y_{\mathrm{ijl}}|X_{\mathrm{ijl}}$. This is true only when the two intervention groups have the same missingness mechanisms and have the same baseline covariate effects on outcome in the outcome model. Therefore, assuming CDM, the baseline covariate adjusted cluster-level analysis is consistent only if the two intervention groups have the same covariate effects on outcome in the outcome model and the same missingness mechanisms. We also note that with no missing data $\mu_{x11}=\mu_{x21}=\mu_{x}$, hence, equation (10) guarantees that the adjusted cluster-level analysis, which assumes that the covariate effect on outcome is the same in both groups, is unbiased, regardless of whether the covariate effect is the same in the intervention groups.

The variance of the estimator (9) can be written as (see Appendix 2 for derivation)

(11) $$\text{Var}\left({\hat{\theta }}_{\text{adj}}^{\text{obs}}\right)=\sum_{i=1}^{2}\frac{1}{k_{i}}\left({\left(\beta_{i}-\lambda \right)}^{2}\sigma_{{\bar{x}}_{i1}}^{2}+\sigma_{b}^{2}+\frac{\sigma_{w}^{2}}{\eta_{i}}\right)$$

This shows that when $\beta_{1}=\beta_{2}$ and the missingness mechanisms are the same in the two intervention groups, in order for the estimator (55) to have minimum variance one should replace the unknown *λ* by an estimate of $\beta_{1}=\beta_{2}=\beta$.

### 5.3 LMM using complete records

Let *Z* be the intervention indicator which is zero for control group and is one for intervention group. When it is assumed that the two intervention groups have the same covariate effects on outcome, we fit a LMM with fixed effects of *X* and *Z*, and a random effect for cluster. Then the estimate of the coefficient of *Z* will be the estimated intervention effect accounting for *X*.

If one thinks that the baseline covariate effects on outcome could be different in the two intervention groups and there are missing outcome values, an interaction of *X* and *Z* must be included in the model. This implies that the intervention effect varies with *X*. Then the estimate of the intervention effect at the mean value of *X* is an estimate of the average intervention effect. Let $X^{*}$ denote the empirically centred variable $X-\bar{X}$, where $\bar{X}$ is the mean of X calculated using data from all individuals. If the baseline covariate effects on outcome are assumed to be different in the two groups, we fit a LMM, using complete records, with fixed effects of $X^{*}$, *Z* and their interaction, and a random effect for cluster. The estimate of the coefficient of *Z* will then be the estimated average intervention effect. One may need to account for the centring step in the variance estimation. We will investigate in the simulations whether ignoring this has any negative impact on CI coverage.

In the general theory of LMM, the variances of the fixed effects parameter estimates, which are calculated based on their asymptotic distributions, are known to be underestimated for small sample sizes.^21^ In this paper, we used quantiles from *t*-distribution with DF $k_{1}+k_{2}-2$ rather than the quantiles form the standard normal distribution to construct the confidence interval for the intervention effect, as this has been used in other papers for individual-level analysis using mixed models for CRTs.^22,23^

## 6 Simulation study

A simulation study was conducted to investigate the performance of unadjusted cluster-level analysis, baseline covariate adjusted cluster-level analysis and LMM using CRA under baseline CDM in outcomes. We also investigated whether there is any gain using MI over CRA. The average estimate of intervention effect, its average estimated SE and coverage probability were calculated and compared. We considered balanced CRTs, where the two intervention groups have equal number of clusters $\left(k_{i}=k\right)$ and constant cluster size $\left(m_{\mathrm{ij}}=m\right)$.

### 6.1 Data generation and analysis

For each individual in the study a single covariate value *X* was generated independently as X~N(0,1). Since $\sigma_{x}^{2}=1$, we can write the coefficient of *X* in equation (1) as $\beta_{i}=\tau_{i}\sigma_{y}$, where $\sigma_{y}^{2}$ is the total variance of *Y* within each intervention group and *τ_i_* is the correlation coefficient between *Y* and *X* in intervention group *i*. We fixed $\sigma_{y}^{2}=100,\alpha_{1}=20$ and $\alpha_{2}=25$. Then the outcome *Y* was generated using the model

$$Y_{\mathrm{ijl}}=\alpha_{i}+\tau_{i}\sigma_{y}X_{\mathrm{ijl}}+\delta_{\mathrm{ij}}+\varepsilon_{\mathrm{ijl}}$$

where $\delta_{\mathrm{ij}}\sim N(0,\rho \sigma_{y}^{2})$ and $\varepsilon_{\mathrm{ijl}}\sim N(0,(1-\tau_{i}^{2}-\rho )\sigma_{y}^{2})$. We chose the cluster size *m* = 30 for each cluster. Parameters that were varied in generating the data include the number of clusters in each group, k=(5,10,20,30) and the unconditional ICC, ρ=(0.001,0.05,0.1). The missing data indicators *R_ijl_* under CDM assumption were generated, independently for each individual, according to a logistic regression model

$$\text{logit}(R_{\mathrm{ijl}}=0|Y_{\mathrm{ij}},X_{\mathrm{ij}})=\varphi_{i0}+\varphi_{i1}X_{\mathrm{ijl}}$$

The intercept $\varphi_{i0}$ and slope $\varphi_{i1}$ were chosen so that ${\text{E}}_{\mathrm{jl}}(R_{\mathrm{ijl}})=p_{i}$, where *p_i_* is the desired proportion of observed values in intervention group *i*. The degree of correlation between missingness and baseline covariate depends on the value of $\varphi_{i1}$. We used $\varphi_{11}=\varphi_{21}=1$, which gives the odds ratio for having a missing outcome (Y) is 2.72 associated with a one unit increase in the covariate (X) value. Missing data indicators were then imposed to each generated complete data to get the incomplete data.

Four possible scenarios were considered:

1. $\varphi_{10}=\varphi_{20}=-1$ and $\tau_{1}=\tau_{2}=0.5$: missingness mechanism is the same between the intervention groups and there is no interaction between intervention group and baseline covariate in the outcome model.
2. $\varphi_{10}=-1,\;\varphi_{20}=0.5$ and $\tau_{1}=\tau_{2}=0.5$: missingness mechanism is different between the intervention groups and there is no interaction between intervention group and baseline covariate in the outcome model.
3. $\varphi_{10}=\varphi_{20}=-1$ and $\tau_{1}=0.4,\;\tau_{2}=0.6$: missingness mechanism is the same between the intervention groups and there is an interaction between intervention group and baseline covariate in the outcome model.
4. $\varphi_{10}=-1,\;\varphi_{20}=0.5$ and $\tau_{1}=0.4,\;\tau_{2}=0.6$: missingness mechanism is different between the intervention groups and there is an interaction between intervention group and baseline covariate in the outcome model.

In the first and third scenarios, there was 30% missing outcomes in both the intervention groups. In the second and fourth scenarios, there was 30% missing outcomes in the control group and 60% missing outcomes in the intervention group. Each generated incomplete dataset was then analysed using unadjusted cluster-level analysis, baseline covariate adjusted cluster-level analysis and LMM using complete records. We included the interaction between intervention and covariate into the LMM in the third and fourth scenarios, where the two intervention groups have different covariate effects on outcome in the data-generating model for outcome.

The R package jomo^24^ was used to multiply impute each generated incomplete dataset using MI with number of imputations 20. A random intercept LMM was used as the imputation model so that the imputation model was correctly specified. We used 200 burn-in iterations and 10 iterations between two successive draws after examining, respectively, the convergence of the posterior distributions of the parameters estimates of the imputation model and the plots of their autocorrelation functions. The completed datasets were then analysed using LMM. An interaction between intervention and baseline covariate was included in both the imputation model and the analysis model when the two intervention groups have different covariate effects on outcome in the data-generating model. We always used restricted maximum likelihood estimation method to fit the LMM. The Wald *t*-test with adjusted DF, given in equation (3), with $\nu_{\text{com}}=2(k-1)$ was used to test the null hypothesis of intervention effect. We had maximum 50 convergence warnings in 10,000 simulations when LMM was fitted using the R package lme4.^25^

### 6.2 Results

Empirical average estimates of intervention effect, average estimated SEs and coverage probabilities of nominal 95% confidence interval over 10,000 simulation runs for each of the four scenarios are presented in Tables 1 to 4, respectively.

**Table 1.** Simulation results-missingness mechanism is the same between the intervention groups and there is no interaction between intervention and baseline covariate in the data-generating model for outcome. Empirical average estimates of intervention effect, average estimated SEs and coverage probabilities of nominal 95% confidence interval over 10,000 simulation runs for unadjusted cluster-level analysis (CL(unadj)), baseline covariate adjusted cluster-level analysis (CL(adj)) and linear mixed model (LMM), using CRA and MI. Monte Carlo errors for average estimates and average estimated SEs are all less than 0.023 and 0.016, respectively. The true value of the intervention effect is 5.

		Average Estimate				Average estimated SE				Coverage (%)			
*ρ*	*k*	CL(unadj)	CL(adj)	LMM	MI	CL(unadj)	CL(adj)	LMM	MI	CL(unadj)	CL(adj)	LMM	MI
0.1	5	4.98	4.99	4.99	4.98	2.31	2.21	2.23	2.19	95.2	95.1	95.2	96.3
	10	5.01	4.98	5.00	4.99	1.66	1.59	1.60	1.59	95.1	95.3	95.3	95.5
	20	4.99	4.99	4.99	4.99	1.18	1.14	1.14	1.14	94.9	95.0	94.9	94.8
	30	5.01	5.00	5.01	5.01	0.97	0.93	0.93	0.93	95.0	95.0	94.9	95.0
0.05	5	5.00	4.98	5.00	5.00	1.88	1.76	1.78	1.76	95.2	95.1	95.6	96.2
	10	5.01	5.00	5.01	5.01	1.35	1.28	1.28	1.26	95.1	95.2	95.1	95.4
	20	5.01	5.00	5.01	5.01	0.96	0.91	0.91	0.90	95.0	95.0	95.1	95.0
	30	4.99	4.99	4.99	4.99	0.79	0.75	0.74	0.74	95.0	95.0	95.0	95.0
0.001	5	4.98	4.98	4.99	4.99	1.34	1.18	1.31	1.35	95.2	95.1	96.2	99.6
	10	5.01	5.00	5.01	5.01	0.96	0.85	0.90	0.93	95.1	95.1	96.8	97.8
	20	4.99	4.99	5.00	5.00	0.69	0.61	0.63	0.64	94.8	94.9	96.2	96.7
	30	5.00	5.00	5.00	5.00	0.56	0.50	0.51	0.52	95.1	95.3	96.2	96.8

When the missingness mechanism is the same between the intervention groups and there is no interaction between intervention and baseline covariate in the outcome model, both the unadjusted and adjusted cluster-level analyses gave unbiased estimates of intervention effect with coverage probabilities very close to the nominal level (see Table 1). However, these two methods gave biased estimates of intervention effect if the two intervention groups had either different missingness mechanisms or there was an interaction between intervention and covariate in the outcome model or both (see Tables 2 to 4). In scenario 2, (two-stage) adjusted cluster-level analysis was very slightly downwardly biased (see Table 2). Under scenario 2, the two intervention groups have the same covariate effects $\left(\beta_{1}=\beta_{2}\right)$ but the missingness mechanism is different between the intervention groups, implying $\mu_{x11}\neq \mu_{x21}$. However, although $R_{\mathrm{ijl}}⊥⊥Y_{\mathrm{ijl}}|X_{\mathrm{ijl}},Z_{i},R_{\mathrm{ijl}}\neg ⊥⊥Y_{\mathrm{ijl}}|X_{\mathrm{ijl}}$, where *Z_i_* is the intervention indicator. Therefore, the estimate of regression coefficient (λ) of the first-stage analysis using CRA was biased as the regression model was fitted without considering *Z_i_*, the intervention indicator. Consequently, the second-stage analysis gave slightly biased estimates of intervention effect. These results support our derived conditions explained in Sections 5.1 and 5.2, respectively, for unadjusted and adjusted cluster-level analyses to be unbiased using CRA, where we showed that these two methods are unbiased only if the missingness mechanism is the same between the intervention groups and there is no interaction between intervention and baseline covariate in the data-generating model for the outcome. These results also imply that cluster mean imputation, as proposed by Taljaard et al.^8^ (described in Section 4.2), is not valid under CDM assumption unless the two intervention groups have the same missingness mechanisms and there is no interaction between intervention and baseline covariate in the outcome model. The bias in average intervention effect estimates could be in either direction. But, in this paper, we always have downward bias in the reported intervention effect estimates. This is because we considered a positive correlation between baseline covariate and outcome in the data generation process, and a positive association between baseline covariate and probability of missingness in outcomes. As a result, a large value of outcome has higher chance of being missing compared to a low value of outcome. In our simulations the degree of bias was high if the two intervention groups had different covariate effects on outcome and it goes up if, in addition, the two intervention groups have different missingness mechanisms (see Tables 3 and 4). LMM and MI gave unbiased estimates of intervention effect under all the four considered scenarios, provided that an interaction of intervention and baseline covariate was included in the model to allow for different covariate effects on outcome in the two intervention groups (scenario 3 and 4).

**Table 2.** Simulation results-missingness mechanism is different between the intervention groups and there is no interaction between intervention and baseline covariate in the data-generating model for outcome. Empirical average estimates of intervention effect, average estimated SEs and coverage probabilities of nominal 95% confidence interval over 10,000 simulation runs for unadjusted cluster-level analysis (CL(unadj)), baseline covariate adjusted cluster-level analysis (CL(adj)) and linear mixed model (LMM), using CRA and MI. Monte Carlo errors for average estimates and average estimated SEs are all less than 0.025 and 0.017, respectively. The true value of the intervention effect is 5.

		Average Estimate				Average estimated SE				Coverage (%)			
*ρ*	*k*	CL(unadj)	CL(adj)	LMM	MI	CL(unadj)	CL(Adj)	LMM	MI	CL(unadj)	CL(Adj)	LMM	MI
0.1	5	3.83	4.94	5.01	5.01	2.44	2.32	2.34	2.28	93.2	95.1	95.2	97.0
	10	3.81	4.94	5.03	5.03	1.76	1.67	1.68	1.66	89.9	95.4	95.2	95.5
	20	3.78	4.91	5.00	4.99	1.25	1.19	1.19	1.19	84.2	94.9	94.8	94.8
	30	3.79	4.93	5.01	5.01	1.02	0.98	0.98	0.98	79.1	95.4	95.3	95.4
0.05	5	3.77	4.90	4.98	4.98	2.04	1.90	1.94	1.92	91.7	94.9	95.7	98.3
	10	3.78	4.90	5.00	4.99	1.48	1.38	1.38	1.36	87.5	95.0	95.0	95.8
	20	3.76	4.92	4.98	4.98	1.05	0.98	0.98	0.97	79.4	95.2	95.1	95.1
	30	3.77	4.92	4.99	4.99	0.86	0.80	0.80	0.80	70.7	94.8	94.6	94.7
0.001	5	3.77	4.89	5.00	5.00	1.58	1.39	1.54	1.60	89.4	95.1	98.3	99.7
	10	3.76	4.89	4.99	4.98	1.14	1.01	1.06	1.10	82.1	95.0	97.3	98.5
	20	3.78	4.91	5.00	5.00	0.81	0.72	0.74	0.76	68.8	95.2	96.4	97.3
	30	3.78	4.92	5.00	5.00	0.66	0.59	0.60	0.61	56.1	94.9	95.8	96.5

**Table 3.** Simulation results-missingness mechanism is the same between the intervention groups and there is an interaction between intervention and baseline covariate in the data-generating model for outcome. Empirical average estimates of intervention effect, average estimated SEs and coverage probabilities of nominal 95% confidence interval over 10,000 simulation runs for unadjusted cluster-level analysis (CL(unadj)), baseline covariate adjusted cluster-level analysis (CL(adj)) and linear mixed model (LMM), using CRA and MI. Monte Carlo errors for average estimates and average estimated SEs are all less than 0.024 and 0.016, respectively. The true value of the intervention effect is 5.

		Average Estimate				Average estimated SE				Coverage (%)			
*ρ*	*k*	CL(unadj)	CL(adj)	LMM	MI	CL(unadj)	CL(Adj)	LMM	MI	CL(unadj)	CL(Adj)	LMM	MI
0.1	5	4.46	4.44	4.97	4.97	2.31	2.22	2.25	2.22	94.3	94.3	95.0	96.4
	10	4.50	4.49	5.01	5.02	1.66	1.59	1.61	1.60	93.7	93.6	94.7	94.8
	20	4.48	4.48	5.00	5.00	1.19	1.14	1.15	1.15	92.5	92.6	94.9	94.9
	30	4.49	4.49	5.00	5.00	0.97	0.93	0.94	0.94	91.3	91.2	94.7	94.7
0.05	5	4.45	4.43	4.96	4.97	1.88	1.76	1.81	1.80	94.0	93.7	95.3	97.1
	10	4.51	4.49	5.01	5.01	1.36	1.28	1.30	1.29	93.7	93.4	95.0	95.5
	20	4.50	4.50	5.01	5.01	0.97	0.91	0.92	0.92	91.9	91.6	94.8	94.8
	30	4.50	4.50	5.01	5.01	0.79	0.75	0.76	0.75	90.4	89.8	94.6	94.6
0.001	5	4.48	4.46	4.99	4.99	1.34	1.18	1.35	1.39	93.4	93.5	98.1	99.4
	10	4.50	4.49	5.02	5.01	0.96	0.85	0.93	0.96	92.3	91.6	96.9	97.9
	20	4.49	4.49	5.00	5.00	0.69	0.61	0.65	0.66	88.9	87.2	96.3	96.8
	30	4.48	4.48	4.99	4.99	0.56	0.50	0.52	0.54	84.9	81.6	95.6	96.3

**Table 4.** Simulation results-missingness mechanism is different between the intervention groups and there is an interaction between intervention and baseline covariate in the data-generating model for outcome. Empirical average estimates of intervention effect, average estimated SEs and coverage probabilities of nominal 95% confidence interval over 10,000 simulation runs using unadjusted cluster-level analysis (CL(unadj)), baseline covariate adjusted cluster-level analysis (CL(Adj)) and linear mixed model (LMM), using CRA and MI. Monte Carlo errors for average estimates and average estimated SEs are all less than 0.025 and 0.018, respectively. The true value of the intervention effect is 5.

		Average Estimate				Average estimated SE				Coverage (%)			
*ρ*	*k*	CL(unadj)	CL(adj)	LMM	MI	CL(unadj)	CL(Adj)	LMM	MI	CL(unadj)	CL(Adj)	LMM	MI
0.1	5	3.02	4.09	5.00	5.00	2.44	2.31	2.42	2.37	89.0	93.4	95.7	98.1
	10	3.03	4.10	5.01	5.01	1.76	1.67	1.73	1.71	82.0	93.5	95.8	96.3
	20	3.03	4.11	5.01	5.01	1.25	1.19	1.23	1.23	66.6	88.8	95.6	95.6
	30	3.03	4.11	5.01	5.02	1.02	0.97	1.01	1.01	52.8	85.9	95.2	95.2
0.05	5	3.02	4.10	5.01	5.01	2.05	1.89	2.06	2.04	87.0	93.9	96.5	99.0
	10	3.02	4.10	5.01	5.01	1.47	1.36	1.45	1.44	75.9	90.4	95.7	96.7
	20	3.01	4.08	4.98	4.98	1.05	0.98	1.03	1.03	55.3	84.9	95.8	95.9
	30	3.02	4.10	5.01	5.00	0.86	0.80	0.84	0.84	38.0	81.1	95.6	95.7
0.001	5	3.02	4.07	4.99	4.99	1.57	1.37	1.69	1.75	80.4	91.1	98.5	99.8
	10	3.03	4.10	5.00	5.00	1.13	0.99	1.17	1.21	63.0	87.6	97.6	98.7
	20	3.02	4.10	5.00	5.00	0.81	0.71	0.81	0.84	33.4	77.7	97.0	97.7
	30	3.01	4.10	5.00	5.00	0.66	0.58	0.66	0.68	16.7	67.9	96.5	97.1

The LMM and MI had similar empirical average estimated SEs of the intervention effect estimates. The LMM gave coverage probabilities close to nominal level except for very small *ρ* and small *k*, where it showed slightly overcoverage. However, while LMM with $\nu_{\text{com}}$ gave good coverage, MI using $\nu_{\text{adj}}$ gave overcoverage, and this can be attributed to it using a smaller DF. The average estimates of $\nu_{\text{adj}}$, used by MI, over 10,000 simulations runs and $\nu_{\text{com}}$ for scenario 4 are presented in Table 5. Results showed that the estimates of $\nu_{\text{adj}}$ are smaller compared to $\nu_{\text{com}}$.

**Table 5.** Comparison between the complete data DF $\left(\nu_{\text{com}}\right)$ and the average estimates of adjusted DF $\left(\nu_{\text{adj}}\right)$, over 10,000 simulation runs, used by MI, when the two intervention groups have different missingness mechanisms and different covariate effects on outcome in the data-generating model for outcome (scenario 4). The last two columns show the upper 2.5% points of the *t-*distribution with $\nu_{\text{com}}$ and $\nu_{\text{adj}}$ DF, respectively.

*ρ*	*k*	$\nu_{\text{com}}$	$\nu_{\text{adj}}$	$t_{\nu_{\text{com}}}(0.025)$	$t_{\nu_{\text{adj}}}(0.025)$
0.1	5	8	4.58	2.31	2.64
	10	18	11.72	2.10	2.18
	20	38	25.71	2.02	2.06
	30	58	38.74	2.00	2.02
0.05	5	8	3.92	2.31	2.80
	10	18	9.64	2.10	2.24
	20	38	20.61	2.02	2.08
	30	58	30.18	2.00	2.04
0.001	5	8	3.12	2.31	3.11
	10	18	7.12	2.10	2.36
	20	38	13.73	2.02	2.14
	30	58	19.01	2.00	2.09

DF: degrees of freedom.

## 7 Discussion and conclusion

In this paper, we aimed to investigate the validity of the unadjusted and adjusted cluster-level analyses, and LMM for analysing CRTs, where the outcomes are continuous and only outcomes are missing under CDM assumption. We used CRA and MI for handling the missing outcomes. The contributions of the paper can be summarised as follows:

First, we found that both the unadjusted and adjusted cluster-level analyses are in general biased using CRA unless there is no interaction between intervention and baseline covariate in the data-generating model for outcome and the missingness mechanism is the same between the interventions groups, which is arguably unlikely to hold in practice. Cluster-level analysis is used by many researchers to analyse CRTs because of its simplicity. We therefore caution researchers that these methods may commonly give biased inferences in CRTs with missing outcomes. However, we note that these two methods are unbiased with full data, even when there is an interaction between baseline covariate and intervention in the true data-generating model for outcome.

Second, cluster mean imputation has been previously recommended as a valid approach for handling missing outcomes in CRTs. We found that cluster mean imputation gave invalid inferences under CDM assumption unless missingness mechanism is the same between the intervention groups and there is no interaction between intervention and baseline covariate in the data-generating model for outcome.

Third, the LMM using CRA gave unbiased estimates of intervention effect regardless of whether missingness mechanisms are the same or are different between the intervention groups and whether there is an interaction between intervention and baseline covariate in the data-generating model for the outcome, provided that an interaction between intervention and baseline covariate was included in the model when such interaction exists in truth.

Finally, we compared the results of LMM using CRA with the results of MI. As expected, we found that MI gave unbiased intervention effects estimates regardless of whether missingness mechanisms are the same or are different in the two intervention groups and whether there is an interaction between intervention and baseline covariate. The LMM and MI had similar empirical SEs of the estimates of intervention effects. However, MI using adjusted DF estimates gave overcoverage for the nominal 95% confidence interval. This is due to underestimation of adjusted DF used by MI compared to complete data DF. Groenwold et al.^20^ showed that there is little to be gained by using MI over LMM in the absence of auxiliary variables. Moreover, when missingness is confined to outcomes, LMMs fitted using maximum likelihood are fully efficient and valid under MAR.

Throughout this paper, we have assumed CDM mechanism in a continuous outcome, which is an example of MAR as our baseline covariate was fully observed. In practice, we cannot identify on the basis of the observed data which missingness assumption is appropriate.^14,26^ Therefore, sensitivity analyses should be performed^26^ (Ch. 10) to explore whether our inferences are robust to the primary working assumption regarding the missingness mechanism. Furthermore, we focused on studies with only one individual-level covariate; the methods described can be extended for more than one covariate.

In conclusion, in the absence of auxiliary variables, LMM using complete records can be recommended as the primary analysis approach for CRTs with missing outcomes if one is willing to make baseline CDM assumption for outcomes.

## Appendix 1. Unadjusted cluster-level analysis using complete records

The mean of the observed outcomes in a particular cluster can be written as

$$\begin{array}{l} {\bar{Y}}_{ij}^{\text{obs}}=\frac{1}{\sum_{l}^{m_{ij}}R_{ijl}}\sum_{l=1}^{m_{ij}}R_{ijl}Y_{ijl}\\ =\frac{1}{\sum_{l}^{\;}R_{ijl}}\sum_{l=1}^{m_{ij}}R_{ijl}\left(\alpha_{i}+\beta_{i}X_{ijl}+\delta_{ij}+\epsilon_{ijl}\right)\\ =\alpha_{i}+\beta_{i}\frac{1}{\sum_{l}^{\;}R_{ijl}}\sum_{l=1}^{m_{ij}}R_{ijl}X_{ijl}+\delta_{ij}+\frac{1}{\sum_{l}^{\;}R_{ijl}}\sum_{l=1}^{m_{ij}}R_{ijl}\epsilon_{ijl}\\ =\alpha_{i}+\beta_{i}{\bar{X}}_{ij}^{\text{obs}}+\delta_{ij}+\frac{1}{\sum_{l}^{\;}R_{ijl}}\sum_{l=1}^{m_{ij}}R_{ijl}\epsilon_{ijl} \end{array}$$

where ${\bar{X}}_{\mathrm{ij}}^{\text{obs}}=(1/\sum_{l}R_{\mathrm{ijl}})\sum_{l=1}^{m_{\mathrm{ij}}}R_{\mathrm{ijl}}X_{\mathrm{ijl}}$ is the observed mean of the baseline covariate *X* in the (ij)th cluster. The expected value of ${\bar{X}}_{\mathrm{ij}}^{\text{obs}}$ across the clusters in the *i*th intervention group will be the true mean of *X* among those individuals with observed outcomes. Let $\mu_{\mathrm{xi}1}$ denote the true mean of the baseline covariate *X* in the *i*th intervention group among those individuals with observed outcomes. Then

$$\text{E}({\bar{Y}}_{\mathrm{ij}}^{\text{obs}})=\alpha_{i}+\beta_{i}\mu_{\mathrm{xi}1}+\text{E}(\frac{1}{\sum_{l}R_{\mathrm{ijl}}}\sum_{l=1}^{m_{\mathrm{ij}}}R_{\mathrm{ijl}}\varepsilon_{\mathrm{ijl}})$$

Let $R_{\mathrm{ij}}=(R_{\mathrm{ij}1},R_{\mathrm{ij}2},\ldots ,R_{{\mathrm{ijm}}_{\mathrm{ij}}})$ be the vector of missing data indicators for the (ij)th cluster. Then

(12) E(1∑l Rijl∑l=1mijRijlϵijl)=E[E(1∑l Rijl∑l=1mijRijlϵijl|Rij)]=E[1∑l Rijl∑l=1mijRijlE(ϵijl|Rij)]=0

since *ε_ijl_*'s are independent of *R_ijl_*'s and $\text{E}(\varepsilon_{\mathrm{ijl}})=0$. Therefore, we have

$$\text{E}({\bar{Y}}_{\mathrm{ij}}^{\text{obs}})=\alpha_{i}+\beta_{i}\mu_{\mathrm{xi}1}$$

The variance of ${\bar{Y}}_{\mathrm{ij}}$ can be written as

Var(Y¯ijobs)=βi2Var(X¯ijobs)+σb2+Var(1∑l Rijl∑l=1mijRijlϵijl)=βi2σx¯i12+σb2+Var(1∑l Rijl∑l=1mijRijlϵijl)

where $\sigma_{{x¯}_{i1}}^{2}$ is the variance of the cluster-specific means of X among those with observed outcomes.

Now

(13) Var(1∑lmijRijl∑l=1mijRijlϵijl)=Var[E(1∑l Rijl∑l=1miijRijlϵijl|Rij)]+E[Var(1∑l Rijl∑l=1mijRijlϵijl|Rij)]=0+E[1(∑l Rijl)2∑l=1mijRijlVar(ϵijl|Rij)], using equation (12)=σw2E(1∑l Rijl)=σw2ηi

where $\text{E}(1/(\sum_{l}^{m_{\mathrm{ij}}}R_{\mathrm{ijl}}))=1/\eta_{i}(\text{say})$. Therefore

$$\text{Var}({\bar{Y}}_{\mathrm{ij}}^{\text{obs}})=\beta_{i}^{2}\sigma_{{x¯}_{i1}}^{2}+\sigma_{b}^{2}+\frac{\sigma_{w}^{2}}{\eta_{i}}$$

The observed mean of the *i*th intervention group is calculated as

$${\bar{Y}}_{i}^{\text{obs}}=\frac{1}{k_{i}}\sum_{j=1}^{k_{i}}{\bar{Y}}_{\mathrm{ij}}^{\text{obs}}$$

Then

$$\text{E}({\bar{Y}}_{i}^{\text{obs}})=\alpha_{i}+\beta_{i}\mu_{\mathrm{xi}1}$$

and

$$\text{Var}({\bar{Y}}_{i}^{\text{obs}})=\frac{1}{k_{i}}(\beta_{i}^{2}\sigma_{{x¯}_{i1}}^{2}+\sigma_{b}^{2}+\frac{\sigma_{w}^{2}}{\eta_{i}})$$

The estimator of intervention effect in unadjusted cluster-level analysis based on observed values is given by

$${\hat{\theta }}^{\text{obs}}={\bar{Y}}_{1}^{\text{obs}}-{\bar{Y}}_{2}^{\text{obs}}$$

Then

E(θ^obs)=(α1+β1μx11)−(α2+β2μx21)=(α1+β1μx)−(α2+β2μx)+β1(μx11−μx)−β2(μx21−μx)=μ1−μ2+β1(μx11−μx)−β2(μx21−μx)

and

Var(θ^obs)=1k1(β12σx¯112+σb2+σw2η1)+1k2(β22σx¯212+σb2+σw2η2)=∑i=121ki(βi2σx¯i12+σb2+σw2ηi)

which tends to zero as $\left(k_{1},k_{2}\right)$ tend to infinity.

## Appendix 2. Adjusted cluster-level analysis using complete records

The mean of observed residuals of a particular cluster is given by

ϵ^¯ijobs=1∑lmijRijl∑l=1mijRijlϵ^ijl=1∑l Rijl∑l=1mijRijl(Yijl−Y^ijl)=1∑l Rijl∑l=1mijRijl(αi+βiXijl+δij+ϵijl−γ−λXijl)=αi+(βi−λ)1∑l Rijl∑l=1mijRijlXijl+δij+1∑l Rijl∑l=1mijRijlϵijl−γ=αi+(βi−λ)X¯ijobs+δij+1∑l Rijl∑l=1mijRijlϵijl−γ

Then

$$\text{E}\left({\bar{\hat{\epsilon }}}_{\mathrm{ij}}^{\text{obs}}\right)=\alpha_{i}+\left(\beta_{i}-\lambda \right)\mu_{xi1}-\gamma$$

and

$$\text{Var}\left({\bar{\hat{\epsilon }}}_{\mathrm{ij}}^{\text{obs}}\right)={\left(\beta_{i}-\lambda \right)}^{2}\sigma_{{\bar{x}}_{i1}}^{2}+\sigma_{b}^{2}+\frac{\sigma_{w}^{2}}{\eta_{i}}$$

using the results (12) and (13). The mean of observed residuals of the *i*th intervention group can be written as

$${\bar{\hat{\epsilon }}}_{i}^{\text{obs}}=\frac{1}{k_{i}}\sum_{j=1}^{k_{i}}{\bar{\hat{\epsilon }}}_{\mathrm{ij}}^{\text{obs}}$$

Then

$$\text{E}\left({\bar{\hat{\epsilon }}}_{i}^{\text{obs}}\right)=\alpha_{i}+\left(\beta_{i}-\lambda \right)\mu_{xi1}-\gamma$$

and

$$\text{Var}\left({\bar{\hat{\epsilon }}}_{i}^{\text{obs}}\right)=\frac{1}{k_{i}}\left({\left(\beta_{i}-\lambda \right)}^{2}\sigma_{{\bar{x}}_{i1}}^{2}+\sigma_{b}^{2}+\frac{\sigma_{w}^{2}}{\eta_{i}}\right)$$

The baseline covariate adjusted estimator of intervention effect, based on observed values, is given by

$${\hat{\theta }}_{\text{adj}}^{\text{obs}}={\bar{\hat{\epsilon }}}_{1}^{\text{obs}}-{\bar{\hat{\epsilon }}}_{2}^{\text{obs}}$$

Then

E(θ^adjobs)=(α1+(β1−λ)μx11−γ)−(α2+(β2−λ)μx21−γ)=(α1+β1μx)−(α2+β2μx)+β1(μx11−μx)−β2(μx21−μx)+λ(μx21−μx11)=μ1−μ2+β1(μx11−μx)−β2(μx21−μx)+λ(μx21−μx11)

and

Var(θ^adjobs)=1k1((β1−λ)2σx¯112+σb2+σw2η1)+1k2((β2−λ)2σx¯212+σb2+σw2η2)=∑i=121ki((βi−λ)2σx¯i12+σb2+σw2ηi)

which tends to zero as $\left(k_{1},k_{2}\right)$ tend to infinity.
